# Predictive Value of 5 Early Warning Scores for Critical COVID-19 Patients

**DOI:** 10.1017/dmp.2020.324

**Published:** 2020-09-09

**Authors:** Hai Hu, Ni Yao, Yanru Qiu

**Affiliations:** Emergency Office of West China Hospital, Sichuan University, Sichuan, China; China International Emergency Medical Team, Sichuan, China; Management Office of Emergency Medical Rescue Base of Sichuan University, Sichuan, China; Department of Critical care medicine, West China Hospital, Sichuan University, Sichuan, China; COVID-19 Medical Team (Hubei) of West China Hospital, Sichuan University, Sichuan, China; Oncology Department of Renmin Hospital of Wuhan University (East campus), Wuhan, Hubei, China; COVID-19 Ward of Renmin Hospital of Wuhan University, Wuhan, Hubei, China

**Keywords:** COVID-19, infectious disease medicine, mortality

## Abstract

**Objectives:**

A simple evaluation tool for patients with novel coronavirus disease 2019 (COVID-19) could assist the physicians to triage COVID-19 patients effectively and rapidly. This study aimed to evaluate the predictive value of 5 early warning scores based on the admission data of critical COVID-19 patients.

**Methods:**

Overall, medical records of 319 COVID-19 patients were included in the study. Demographic and clinical characteristics on admission were used for calculating the Standardized Early Warning Score (SEWS), National Early Warning Score (NEWS), National Early Warning Score2 (NEWS2), Hamilton Early Warning Score (HEWS), and Modified Early Warning Score (MEWS). Data on the outcomes (survival or death) were collected for each case and extracted for overall and subgroup analysis. Receiver operating characteristic curve analyses were performed.

**Results:**

The area under the receiver operating characteristic curve for the SEWS, NEWS, NEWS2, HEWS, and MEWS in predicting mortality were 0.841 (95% CI: 0.765-0.916), 0.809 (95% CI: 0.727-0.891), 0.809 (95% CI: 0.727-0.891), 0.821 (95% CI: 0.748-0.895), and 0.670 (95% CI: 0.573-0.767), respectively.

**Conclusions:**

SEWS, NEWS, NEWS2, and HEWS demonstrated moderate discriminatory power and, therefore, offer potential utility as prognostic tools for screening severely ill COVID-19 patients. However, MEWS is not a good prognostic predictor for COVID-19.

The 2019 novel coronavirus (SARS-CoV-2) was discovered following an unexplained viral pneumonia case in Wuhan, China, in December 2019, and was named by the World Health Organization (WHO) on January 12, 2020. Although most patients with novel coronavirus disease 2019 (COVID-19) only suffered cold-like symptoms and recovered without special therapy,^1^ a few patients became dangerously ill.^2^ Previous reports described a mortality rate of 11% to 62% among severely or critically ill patients with COVID-19.^3-6^ The mortality inpatients aged ≥65 y was higher than that in younger patients.^2^ Hitherto, identifying patients with life-threatening illness and enabling them to access early advanced medical intervention may contribute to reducing mortality. Due to the large number of patients with COVID-19 and relatively insufficient medical resources, emergency screening of such patients has become challenging.^7^

Currently, although clinicians use indices such as the white blood cell count,^1^ C-reactive protein concentration,^1^ interleukin,^1^ or d-dimer^6^ to classify COVID-19 cases for severity, these prognostic factors are nonspecific and may involve a delay. Therefore, these indicators were unsuitable for screening, and there is no particular scoring system created for COVID-19 patients. Thus, the second-best choice is applying existing scoring tools. We focused on existing early warning scores (EWSs) to evaluate the prognosis of COVID-19 patients. The EWSs were widely applied in the emergency department (ED) for assisting emergency physicians to predict the risk of deterioration, monitor the evolution of the patient, and make clinical decisions, specifically to promote the safety of the critical patient. So far, many models of EWSs have been developed, such as National Early Warning Score (NEWS), National Early Warning Score 2 (NEWS2), Hamilton Early Warning Score (HEWS), Modified Early Warning Score (MEWS), and Standardized Early Warning Score (SEWS) (see Supplemental Table 1, which is available online). In 2012, the Royal College of Physicians (RCP) proposed NEWS,^8^ whose parameters included heart rate, systolic blood pressure, temperature, respiratory rate, oxygen saturation, supplemental oxygen, and mental status. NEWS has now undergone extensive validation.^9,10^ Five years later, the RCP updated NEWS to NEWS2,^11^ which used the same parameters but altered their weights. HEWS, whose parameters are also the same as those of NEWS but with different weights, was developed in 2015, has successfully completed pilot-testing in the ED setting,^12-14^ and is being used in some medical centers in Canada. MEWS was originally developed in 2000 to facilitate timely recognition of patients with established or impending critical illness.^15^ Its parameters are heart rate, systolic blood pressure, respiratory rate, body temperature, and mental status. Some studies^16-18^ have suggested that MEWS may be suitable for use in the ED and may allow improvement in the quality and safety of care. SEWS was proposed by Paterson’s team^19^ in 2006. Its parameters are respiratory rate, temperature, systolic blood pressure, oxygen saturation, heart rate, and mental status. Paterson et al. illuminated that SEWS correlates with in-hospital mortality.^19^

Currently, the EWS series is widely used in EDs.^20^ Although some studies^21-23^ on clinical therapies for severely ill COVID-19 patients have used EWSs as tools to stratify risk, to our knowledge, no studies comparing the performance of these scores in COVID-19 patients. Thus, the purpose of our study was to evaluate the use of these EWSs as in-hospital mortality indicators for COVID-19 patients.

## METHODS

### Study Design

This was a retrospective analysis of data obtained from an electronic register of COVID-19 patients who visited our ED in early 2020. The data accessed were anonymized.

*The Diagnosis and Treatment Plan of Novel Coronavirus Pneumonia (Version 6)*, which was issued by the National Health Commission of China, was adopted for diagnosis, classification, and treatment of COVID patients (see Supplemental Table 2). The 5 EWSs were applied to the data, and their capacity to predict in-hospital mortality of patients was assessed.

### Study Setting

The emergency medical team, containing more than 100 members, was deployed to Wuhan city during the COVID-19 epidemic and managed the temporary COVID-19 ward independently in a hospital in Wuhan.

The local Institutional Review Committee approved the study and waived the need for obtaining informed consent from the study subjects, owing to the study design. The study complied with the international ethical guidelines for human research, including the Declaration of Helsinki.

### Subjects

**FIGURE 1:**
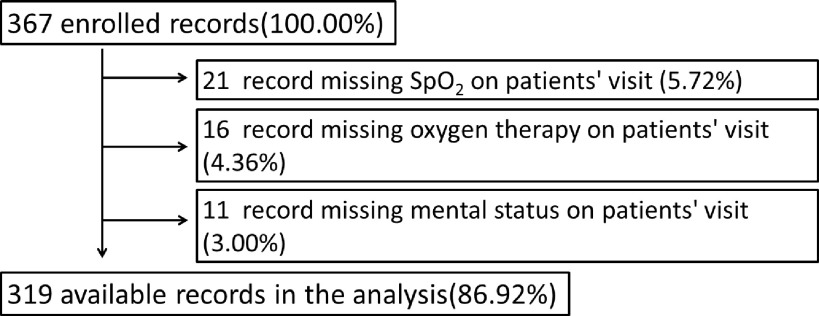
Flow chart of cases exclusion. From all 367 cases, we excluded 21 cases missing information on oxygen saturation, 16 cases missing record on oxygen therapy and 11 cases missing information on mental status. Finally, we analyzed 319 cases.

We enrolled 367 cases aged ≥18 y diagnosed with COVID-19 between January 13 and April 13, 2020. Excluding 48 cases with missing data, 319 were analyzed (Figure 1). The following data were retrieved from the electronic register: basic information (sex, age, diagnosis, and chronic diseases), initial vital signs (body temperature, heart rate, blood pressure, and respiratory rate), consciousness on patients’ visit, oxygen saturation level on patients’ visit, and other variables used to calculate the 5 scoring systems. The outcome was the patient’s death or survival at discharge.

### Statistical Analysis

The data analysis was conducted using the IBM Statistical Program for Social Sciences Statistics 20.0 (SPSS; IBM Corp., Armonk, NY) and MedCalc Statistical Software (Version 18.2.1; MedCalc Software Ltd., Ostend, Belgium). Continuous variables are presented as mean ± standard deviation or median (25%quartile, 75%quartile), as appropriate. Categorical variables are described as composition ratios (%). The comparison of continuous variables used Student’s *t* test or the Mann-Whitney *U* test, and that of categorical variables used the chi-squared test or Fisher’s exact test, as appropriate. Receiver operating characteristic (ROC) curves were used to evaluate the capacities of the 5 scoring systems to predict mortality. The area under the ROC curve (AUC) and its 95% confidence interval (95% CI) were calculated. An AUC > 0.75 was regarded as indicating a test of acceptable clinical value and an AUC > 0.97 as a test of high clinical value.^24^ The AUCs were compared using the DeLong test,^25^ and the best demarcation point of each scoring scale was determined as the maximum value of the Youden index. Then, the corresponding accuracy, sensitivity, specificity, positive predictive value (PPV), and negative predictive value (NPV) corresponding to the best cutoff point of each score were calculated. Finally, we calculated the worst Sequential Organ Failure Assessment (SOFA) score during the first 24 h. And we analyzed the correlation between the worst SOFA score and each EWS through Spearman’s rank correlation test. For all analyses, a *P* value < 0.05 was considered to indicate statistical significance.

## RESULTS

### Baseline Analysis

The mean age of survivors and nonsurvivors was 53.93 ± 15.32 and 71.63 ± 13.16 y, respectively, and this difference was significant (*P* < 0.05).The length of hospital stay of survivors, which refers to the duration from admission to discharge, was 17[9.25, 29] d. And the length of hospital stay of nonsurvivors, which refers to the duration from admission to death, was 4.5[3, 11.25] d. These 2 groups differed significantly with respect to gender, respiratory rate, pulse rate, heart rate, peripheral oxygen saturation, consciousness, and NEWS, NEWS2, HEWS, MEWS, and SEWS scores (*P* < 0.05). Details are shown in Table 1.

**TABLE 1:** Comparison of the Baseline Characteristics of Survivors and Non-survivors

Variable	Survivors *N* = 279	Non-survivors *N* = 40	*P*-Value
Male (number)	120(43.01%)	30(75.00%)	0.000*
Age (years)	53.93±15.32	71.63±13.16	0.000*
RR (/min)	20[18,21]	21[18.75,27.25]	0.002*
Temperature (°C)	36.77±0.63	36.79±0.61	0.766
SBP (mmHg)	128.95±15.9	134.36±24.55	0.058
DBP (mmHg)	77.08±11.73	78.23±13.54	0.554
SpO_2_ (%)	95.5[92,98]	90[88,92.75]	0.000*
HR (/min)	83.8±19.49	90.77±16.51	0.026*
GCS	15[range 15-15]	15[range 10-15]	0.005*
AVPU			
A	279(100%)	36(90.00%)	0.000*
V, P, U	0(0%)	4(10.00%)	
Underlying disease			
Diabetes	21(7.53%)	4(10.00%)	0.534
Hypertension	58(20.79%)	17(42.50%)	0.005*
Cardiovascular disease	23(8.24%)	8(20.00%)	0.039*
Chronic pulmonary disease	3(1.08%)	4(10.00%)	0.006*
Cerebral vascular disease	1(0.36%)	3(7.50%)	0.007*
Malignant tumor	11(3.94%)	3(7.50%)	0.397
Patients under monitoring	92(32.97%)	33(82.50%)	0.000*
Mechanical ventilation	4(1.43%)	13(32.50%)	0.000*
Renal replacement therapy	2(0.72%)	1(2.50%)	0.332
SEWS (score)	4[3,4.5]	6[5,8]	0.000*
NEWS (score)	4[2,5]	7[5,8]	0.000*
NEWS2 (score)	4[2,5]	7[5,8]	0.000*
HEWS (score)	2[1,3]	4[3,6.25]	0.000*
MEWS (score)	1[1,2]	2[1,3]	0.000*

Abbreviations: PR, pulse rate; HR, heart rate; RR, respiratory rate; SBP, systolic blood pressure; DBP, diastolic blood pressure; MAP, mean arterial pressure; SpO_2_, peripheral oxygen saturation; GCS, Glasgow Coma Scale score; AVPU: AVPU assessment (A, alert; V, respond to verbal commands; P, respond to painful stimuli; U, unconscious); NEWS, National Early Warning Score; NEWS2, National Early Warning Score2; HEWS, Hamilton Early Warning Score; MEWS, Modified Early Warning Score. SEWS, Standardized Early Warning Score

* *P* < 0.05.

### Overall Analysis

**FIGURE 2:**
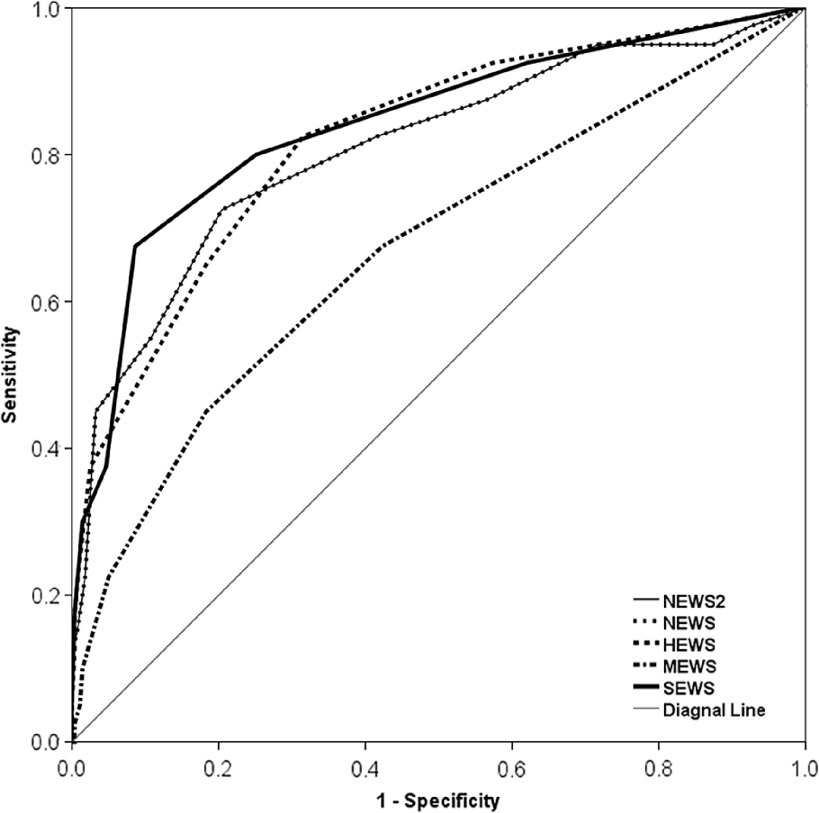
The ROC curves of five EWSs for overall cases ROC: Receiver operating characteristic; NEWS: National Early Warning Score; NEWS2: National Early Warning Score2; HEWS: Hamilton Early Warning Score; MEWS: Modified Early Warning Score. SEWS: Standardized Early Warning Score.

The results of ROC curve analysis were showed in Table 2 and Figure 2. The AUCs of SEWS, HEWS, NEWS, NEWS2, and MEWS were 0.841, 0.821, 0.809, 0.809, and 0.670 (*P* < 0.05). The optimal cutoff values of SEWS, NEWS, NEWS2, HEWS, and MEWS were 7, 10, 10, 8, and 5, respectively (Table 2).

**TABLE 2:** Performance of 5 Scoring Systems in Predicting in-Hospital Mortality of COVID-19 Patients

Models	AUC	95% CI	*P*-Value	Cutoff Value	Sen. (%)	Spe. (%)	Accuracy (%)	PPV	NPV
Overall									
SEWS	0.841	0.765-0.916	0.000*	7	78.95	91.67	90.91	37.50	98.57
NEWS	0.809	0.727-0.891	0.000*	10	75.00	89.07	88.71	15.00	99.28
NEWS2	0.809	0.727-0.891	0.000*	10	75.00	89.07	88.71	15.00	99.28
HEWS	0.821	0.748-0.895	0.000*	8	80.00	89.64	89.34	20.00	99.28
MEWS	0.670	0.573-0.767	0.001*	5	50.00	88.42	87.46	10.00	98.57
⩾65 y									
SEWS	0.808	0.714-0.903	0.000*	7	87.50	78.22	78.90	24.14	98.75
NEWS	0.829	0.734-0.924	0.000*	8	85.71	82.11	82.57	41.38	97.50
NEWS2	0.829	0.734-0.924	0.000*	8	85.71	82.11	82.57	41.38	97.50
HEWS	0.809	0.722-0.896	0.000*	6	90.00	79.80	80.73	31.03	98.75
MEWS	0.650	0.527-0.773	0.017*	4	75.00	77.23	77.06	20.69	97.50
<65 y									
SEWS	0.893	0.756-0.999	0.000*	7	80.00	96.59	96.19	36.36	99.50
NEWS	0.803	0.630-0.976	0.001*	10	62.50	97.03	95.71	45.45	98.49
NEWS2	0.803	0.630-0.976	0.001*	10	62.50	97.03	95.71	45.45	98.49
HEWS	0.820	0.638-0.999	0.000*	8	50.00	97.47	94.76	54.55	96.98
MEWS	0.704	0.530-0.877	0.023*	5	50.00	96.08	94.76	27.27	98.49

Abbreviations: AUC, area under the curve of the receiver operating characteristic; 95% CI, 95% confidence interval; Sen., sensitivity; Spe., specificity; PPV, positive predictive value; NPV, negative predictive value; LR+, likelihood ratio positive; LR-, likelihood ratio negative; NEWS, National Early Warning Score; NEWS2, National Early Warning Score2; HEWS, HamiltonEarly Warning Score; MEWS, Modified Early Warning Score; SEWS, Standardized Early Warning Score; N/A, Not available because the denominator is zero.

* *P* < 0.05.

Based on the best Youden index, an optimum cutoff value was used to predict in-hospital mortality using each EWS. The cutoff values for each score, together with the sensitivity, specificity, PPV, and NPV are shown in Table 2 and Supplemental Table 3.

Pairwise comparisons of the AUCs associated with the 5 EWSs, showed significant differences among 4 pairs, including SEWS versus MEWS, NEWS versus MEWS, NEWS2 versus MEWS and HEWS versus MEWS. There was no statistical difference between other paired values (Table 3).

**TABLE 3:** Pairwise Comparison of AUC of 5 Scoring Systems for Predicting in-Hospital Mortality of COVID-19 Patients

Pairwise Models	△AUC	95% CI	z Statistic	*P*-Value
Overall				
SEWS ~ NEWS	0.0317	-0.0294 to 0.0927	1.017	0.3091
SEWS ~ NEWS2	0.0317	-0.0294 to 0.0927	1.017	0.3091
SEWS ~ HEWS	0.0194	-0.0294 to 0.0682	0.780	0.4356
SEWS ~ MEWS	0.1700	0.0884 to 0.252	4.071	0.0000*
NEWS ~ NEWS2	0.0000	0.000 to 0.000	/	0.9999
NEWS ~ HEWS	0.0123	-0.0404 to 0.0650	0.457	0.6480
NEWS ~ MEWS	0.1390	0.0471 to 0.230	2.966	0.0030*
NEWS2 ~ HEWS	0.0123	-0.0404 to 0.0650	0.457	0.6480
NEWS2 ~ MEWS	0.1390	0.0471 to 0.230	2.966	0.0030*
HEWS ~ MEWS	0.1510	0.0799 to 0.222	4.161	0.0000*
<65 y				
SEWS ~ NEWS	0.0900	-0.00825 to 0.188	1.795	0.0726
SEWS ~ NEWS2	0.0900	-0.00825 to 0.188	1.795	0.0726
SEWS ~ HEWS	0.0731	-0.0247 to 0.171	1.465	0.1428
SEWS ~ MEWS	0.1890	0.0462 to 0.332	2.595	0.0095*
NEWS ~ NEWS2	0.0000	0.000 to 0.000	/	0.9999
NEWS ~ HEWS	0.0169	-0.0306 to 0.0644	0.698	0.4853
NEWS ~ MEWS	0.0989	0.00606 to 0.192	2.088	0.0368*
NEWS2 ~ HEWS	0.0169	-0.0306 to 0.0644	0.698	0.4853
NEWS2 ~ MEWS	0.0989	0.00606 to 0.192	2.088	0.0368*
HEWS ~ MEWS	0.1160	-0.00635 to 0.238	1.858	0.0632
⩾65 y				
SEWS ~ NEWS	0.0207	-0.0605 to 0.102	0.499	0.6175
SEWS ~ NEWS2	0.0207	-0.0605 to 0.102	0.499	0.6175
SEWS ~ HEWS	0.0009	-0.0567 to 0.0584	0.029	0.9766
SEWS ~ MEWS	0.1580	0.0575 to 0.258	3.082	0.0021*
NEWS ~ NEWS2	0.0000	0.000 to 0.000	/	0.9999
NEWS ~ HEWS	0.0198	-0.0580 to 0.0977	0.499	0.6178
NEWS ~ MEWS	0.1790	0.0491 to 0.308	2.704	0.0069*
NEWS2 ~ HEWS	0.0198	-0.0580 to 0.0977	0.499	0.6178
NEWS2 ~ MEWS	0.1790	0.0491 to 0.308	2.704	0.0069*
HEWS ~ MEWS	0.1590	0.0676 to 0.250	3.413	0.0006*

Abbreviations: △AUC, difference between areas under curves; 95% CI, 95% confidence interval; NEWS, National Early Warning Score; NEWS2, National Early Warning Score2; HEWS, Hamilton Early Warning Score; MEWS, Modified Early Warning Score; SEWS, Standardized Early Warning Score.

* *P* < 0.05.

### Subgroup Analysis

**FIGURE 3:**
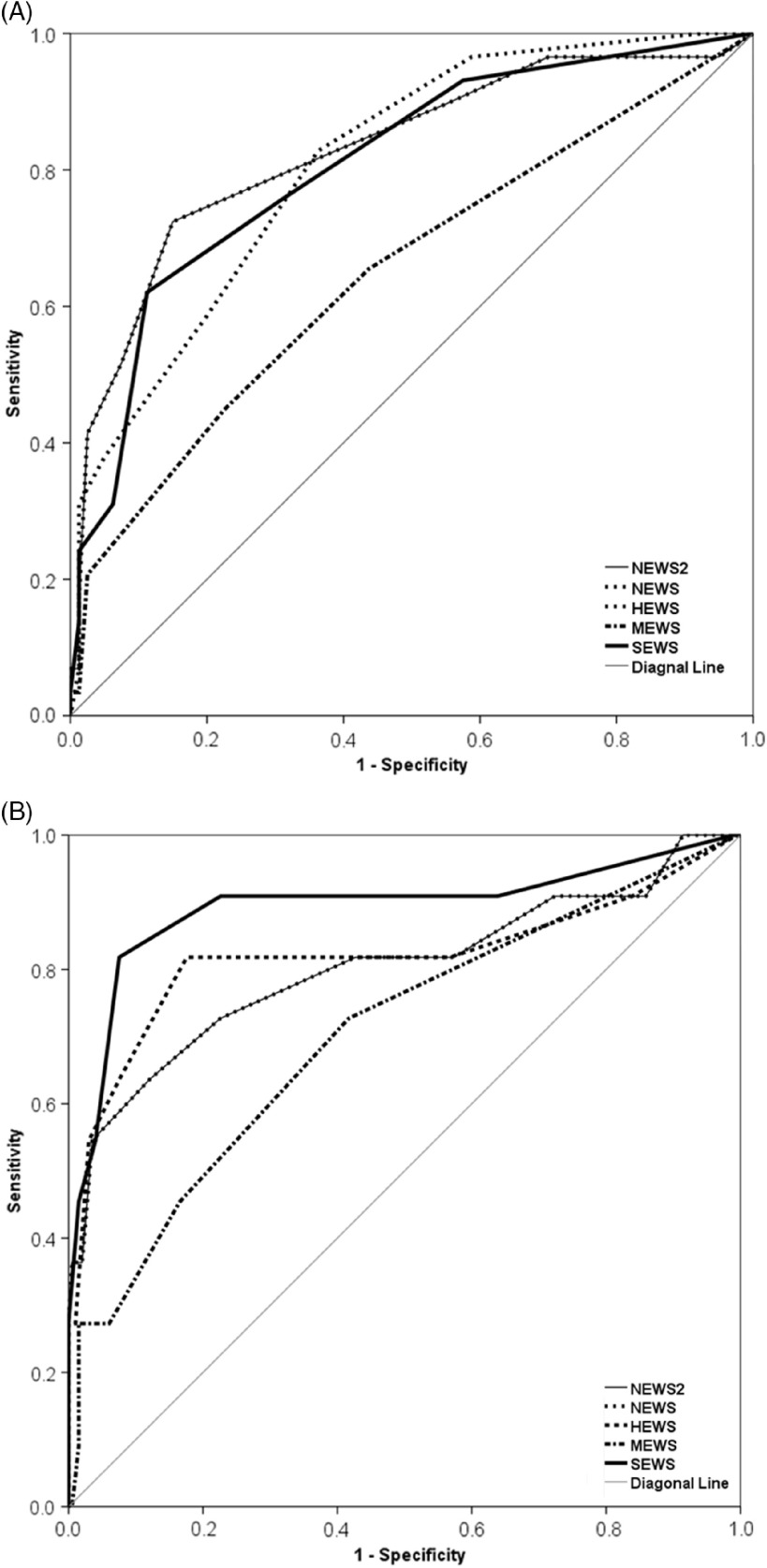
Classification performances of five early warning scores (EWS) for subgroups. (A) The ROC curves of five EWSs in the subgroup of persons aged ≥65 years; (B) The ROC curves of five EWSs in the subgroup of persons aged <65 years. NEWS: National Early Warning Score; NEWS2: National Early Warning Score2; HEWS: Hamilton Early Warning Score; MEWS: Modified Early Warning Score. SEWS: Standardized Early Warning Score.

In the subgroup of age **≥** 65 y, the AUCs of SEWS, NEWS, NEWS2, HEWS, and MEWS were 0.808, 0.829, 0.829, 0.809, and 0.650, respectively. The optimal cutoff values of SEWS, NEWS, NEWS2, HEWS, and MEWS were 7, 8, 8, 6, and 4, respectively (Table 2; Figure 3A). Paired comparisons showed a significant difference between the MEWS score and all other scores. There was no statistical difference between other paired values (Table 3).

In the subgroup of age <65 y, the AUCs of SEWS, NEWS, NEWS2, HEWS, and MEWS were 0.893, 0.803, 0.803, 0.820, and 0.704. The optimal cutoff values of SEWS, NEWS, NEWS2, HEWS, and MEWS were 7, 10, 10, 8, and 5, respectively (Table 2; Figure 3B).

Pairwise comparisons showed significant differences among 2 pairs, including SEWS versus MEWS, NEWS versus MEWS, and NEWS2 versus MEWS. There was no statistical difference between other paired values (Table 3).

### Correlation Between Each EWSs and SOFA

As 45 cases lacked the information needed to calculate the worst SOFA in the first 24 h, we exclude these cases and analyzed 274 cases for the correlation between each EWS and SOFA. The median and quartiles of SOFA score were 4[4, 6]. The Correlation coefficients of SOFA versus SEWS, NEWS, NEWS2, HEWS and MEWS were 0.294, 0.330, 0.330, 0.278, and 0.221, respectively (*P* < 0.05, see Supplemental Table 4).

## DISCUSSION

COVID-19, a novel infectious disease caused by SARS-CoV-2, led to more than 9 million confirmed cases worldwide from outbreak to June 2020. Although most cases suffered mild symptoms, some progressed to viral pneumonia and multi-organ dysfunction. Rapid and accurate identification of severely ill patients would promote the allocation of appropriate medical resources.

The application of scoring systems can facilitate effective evaluation by emergency or critical care physicians to screen severe patients. At present, however, there are no specific scoring systems for the evaluation of COVID-19 patients. Another option is to adopt existing scoring systems that are used for predicting the mortality of severe patients.^26^ EWSs, which are generally used in the patients with sepsis, trauma, and other critical ills, are such scoring systems and are widely used in hospitals to identify patients who are clinically deteriorating.^17,20,27,28^

On the whole, the performance of each EWS was acceptable for screening the COVID-19 patients. However, there are differences in the performance among 5 EWSs.

Overall cases and in both age subgroups the AUC of SEWS was acceptable, demonstrating moderate discriminatory power and potential utility as a predictor of mortality in severe COVID-19 patients. An NPV of 98.57% for SEWS would enable emergency physicians to decisively exclude COVID-19 patients with a SEWS score below 7 from the high-risk group. For COVID-19 patients, the advantage of SEWS is the inclusion of more appropriate parameters, including those that differ between survivors and nonsurvivors in univariate analysis, such as heart rate, respiratory rate, oxygen saturation, and mental status.

The AUCs of NEWS and NEWS2 were 0.809 in overall analysis and a higher NPV of 99.28% suggest that these scores may be better screening tools for emergency physicians. In both age subgroups, the predictive performance of NEWS and NEWS2 was acceptable. Some therapeutic studies of COVID-19 patients^21,22^ have used NEWS or NEWS2 as baseline comparison tools. Although these studies did not evaluate the predictive value of NEWS and NEWS2, the researchers believed that these EWSs could be used as tools for stratification of critical illness.

HEWS performed moderately among the 5 EWSs overall and the subgroup analysis. Its AUC suggest that HEWS is similar to SEWS, NEWS, and NEWS2 for screening COVID-19 patients.

As it does not take into account oxygen saturation (which is very important for COVID-19 patients), it was not surprising that MEWS performed worst in predicting mortality. Therefore, we do not recommend it as a risk stratification tool for severe COVID-19 patients.

We also found that each EWS had only weak positive correlation with SOFA (*P* < 0.05; correlation coefficient 0.221-0.330). In our study, the SOFA includes laboratory testing parameters, which cannot be calculated at the same time as EWS being calculated while the patients just arrived at the hospital. Therefore, the difference of assessing time may be the reason why these 2 variables are only weakly correlated. This thus makes us realize that the advantage of EWSs is likely to lie in the rapid triage of COVID-19. Further study could explore the relationship between EWSs and SOFA at the same scoring time.

We believe this study was the first to explore the practical value of 5 existing EWSs for rapid screening of severe COVID-19 patients. However, its limitations must be acknowledged. First, we used in-hospital mortality as the main outcome, which means all the living patients at time of discharge (discharge criteria see Supplemental Table 2) would be considered a survivor. Although the sudden death after discharge would not be predicted, as no report on sudden death of COVID-19 patients after discharge was found, we inferred that sudden death of COVID-19 patients after discharge is rare. Thus, we considered in-hospital mortality can be used for evaluating the performance of EWSs of identifying severely ill patients to promote the allocation of appropriate medical resources in hospital. In addition, we also used the worst SOFA as the secondary outcome. Nevertheless, further prospective studies of survival analysis could be done for COVID-19 patients.

Second, whether an oxygen supply was given during SpO_2_ measurement may be a confounder. In this study, all SpO_2_ data are from the first record of the patient when they just arrive at the hospital. Although the SpO_2_ measurement at this time is usually on room air or after a short time of oxygen supplying, we cannot confirm that the SpO_2_ values of all patients are measured without oxygen supply due to a retrospective study design. Thus, the prospective study with well-designed should be done to control the condition during SpO_2_ measurement. Third, due to the lack of COVID-19 patients with hypercapnia, our study was unable to distinguish the performance of NEWS and NEWS2. Because the difference between them is that the oxygen saturation scale of NEWS is uniform for all patients, while that of NEWS2 distinguishes patients with hypercapnic respiratory insufficiency. Fourth, we neglected prehospital care because this information was not available. Last, as the study was confined to a single center, it may have been affected by selection bias.

## CONCLUSIONS

The SEWS, NEWS, NEWS2, and HEWS have the potential to be used as tools for screening severely and critically ill COVID-19 patients. Inclusion of these tools in decision strategies could provide a more effective evaluation of mortality rate, thus avoiding delayed medical attention. And MEWS is unsuitable for COVID-19 patients because its performance is inferior to that of other 4 EWSs.

## Supplementary material

For supplementary material accompanying this paper visit https://doi.org/10.1017/dmp.2020.324. click here to view supplementary material
