# Supplementary material for: Predictive Value of 5 Early Warning Scores for Critical COVID-19 Patients
Source: Disaster Med Public Health Prep. 2020 Sep 9:1–8. doi: 10.1017/dmp.2020.324 (PMC7596567; doi:10.1017/dmp.2020.324)
Supplement: Supplementary file 1 [file S1935789320003249sup001.docx]

**Supplementary**

**Supplementary 1**

**Early warning scoring systems**^§^

| **Scoring system** | **parameters** |  |  |  | **Score** |  |  |  |
| --- | --- | --- | --- | --- | --- | --- | --- | --- |
|  |  | **3** | **2** | **1** | **0** | **1** | **2** | **3** |
| **SEWS** | RR | <9 |  |  | 9-20 | 21-30 | 31-35 | >35 |
|  | SpO2 | <85 | 85-89 | 90-92 | 93-100 |  |  |  |
|  | T | <34 | 34-34.9 | 35-35.9 | 36-37.9 | 38-38.9 | >38.9 |  |
|  | SBP | <70 | 71-79 | 80-99 | 100-199 |  | >199 |  |
|  | HR | <30 | 30-39 | 40-49 | 50-99 | 100-109 | 110-129 | >129 |
|  | MS |  |  |  | A | V | P | U |
|  |  |  |  |  |  |  |  |  |
| **NEWS** | HR(/min) | ≤40 |  | 41-50 | 51-90 | 91-110 | 111-130 | >130 |
|  | SBP(mmHg) | ≤90 | 91-100 | 101-110 | 111-219 |  |  | ≥220 |
|  | T(°C) | ≤35 |  | 35.1-36 | 36.1-38 | 38.1-39 | ≥39.1 |  |
|  | RR(/min) | ≤8 |  | 9-11 | 12-20 |  | 21-24 | ≥25 |
|  | SpO_2_(%) | ≤91 | 92-93 | 94-95 | ≥96 |  |  |  |
|  | O_2_ supply |  | Yes |  | No |  |  |  |
|  | MS |  |  |  | A |  |  | V,P,U |
|  |  |  |  |  |  |  |  |  |
| **NEWS2** | HR | ≤40 |  | 41-50 | 51-90 | 91-110 | 111-130 | >130 |
|  | SBP | ≤90 | 91-100 | 101-110 | 111-219 |  |  | ≥220 |
|  | T | ≤35 |  | 35.1-36 | 36.1-38 | 38.1-39 | ≥39.1 |  |
|  | RR | ≤8 |  | 9-11 | 12-20 |  | 21-24 | ≥25 |
|  | SpO_2_ |  |  |  |  |  |  |  |
|  | Scale1 | ≤91 | 92-93 | 94-95 | ≥96 |  |  |  |
|  | Scale2* | ≤83 | 84-85 | 86-87 | 88-92  ≥93 air | 93-94  Oxygen | 95-96  Oxygen | ≥97  Oxygen |
|  | O_2_supply |  | Yes |  | No |  |  |  |
|  | MS |  |  |  | A |  |  | V,P,U |
|  |  |  |  |  |  |  |  |  |
| **HEWS** | HR |  | ≤40 | 41-50 | 51-100 | 101-110 | 111-130 | >130 |
|  | SBP | <71 | 71-90 |  | 91-170 |  | 171-200 | >200 |
|  | T | ≤35 |  | 35.1-36 | 36.1-37.9 | 38-39 | ≥39.1 |  |
|  | RR | <8 | 8-13 |  | 14-20 |  | 21-30 | >30 |
|  | SpO_2_ | <85 |  | 85-92 | >92 |  |  |  |
|  | O_2_supply |  |  |  | No | ≤5 L/min or <50% |  | >5 L/min or ≥50% |
|  | MS |  | delirium |  | A | V | P | U |
|  |  |  |  |  |  |  |  |  |
| **MEWS** | HR |  | <40 | 41-50 | 51-100 | 101-110 | 111-129 | >129 |
|  | SBP | <70 | 71-80 | 81-100 | 101-199 |  | >199 |  |
|  | T |  | <35 |  | 35-38.4 |  | >38.4 |  |
|  | RR |  | <9 |  | 9-14 | 15-20 | 21-29 | >29 |
|  | MS |  |  |  | A | V | P | U |

NEWS: National Early Warning Score; NEWS2: National Early Warning Score2; HEWS: Hamilton Early Warning Score; MEWS: Modified Early Warning Score; SEWS: Standardized Early Warning Score; HR: Heart rate; SBP: Systolic blood pressure; T: Temperature; RR: Respiratory rate;SpO2: Oxygen saturation; O_2_supply:Supplemental oxygen; MS: Mental status; A: alert; V: response to verbal command; P: response to pain; U: unresponsive;

*In patients with hypercapnia respiratory insufficiency, scale 2 should be used to weight the oxygen saturation score

§The total score of each EWS is equal to the sum of the scores of each parameter.

**Supplementary 2**

**The criterion*** **of COVID-19 in the study**

| **Criteria** | **Details** |
| --- | --- |
| **Diagnostic Criteria** | Patients with an epidemiological history or clinical manifestations meet one of the following two conditions:  1. Real-time fluorescent RT-PCR indicates positive for nucleic acid of the 2019 novel coronavirus (SARS-Cov-2)  2. Viral gene sequence is highly homologous to known SARS-Cov-2 |
| **Classification Criteria** | **Minor**  The confirmed patient has mild symptoms，without radiography results of pneumonia. |
|  | **Common**  The confirmed patient has fever and respiratary symptoms，with radiography results of pneumonia. |
|  | **Severe**  the confirmed COVID-19 patients who meet any one of the follows:  1. Shortness of breath, with RR≥ 30 times/min  2. Oxygen saturation ≤ 93%  3. Oxygenation index≤300mmHg^§^  4. Chest radiographic images showed that the lesion progressed more than 50% within 24-48 hours |
|  | **Critical**  the severe confirmed COVID-19 patients who meet any one of the follows  1. Respiratory failure needs mechanical ventilation  2. Shock  3. Combining other organ failure needs ICU admission |
| **Discharge Criteria** | Those who meet all the following criteria can be discharged.  1) Normal body temperature > 3 days;  2) Symptoms of respiratory system are improved obviously;  3) The pulmonary infection is absorbed obviously, tested by chest imaging  4) Of respiratory tract samples, consecutive twice nuclei acid tests is both negative. (Sampling interval≥ 24 hours). |

COVID-19: novel coronavirus disease; RR: respiratory rate; ICU: intensive care unit

*The criterion is followed the *Diagnosis and Treatment Plan of Novel Coronavirus*, which issued by issued by the National Health Commission of China and can be download in http://www.nhc.gov.cn. The original was issued in Chinese, so we translated its gist into English.

§High altitude areas need correction

**Supplementary 3**

**The performance of five scoring systems in predicting in-hospital mortality of COVID-19 patients**

| **Models** | **Cutoff value** | **Sen.**  **(%)** | **Spe.(%)** | **Youden Index** | **Accuracy**  **(%)** | **PPV** | **NPV** |
| --- | --- | --- | --- | --- | --- | --- | --- |
| SEWS | 1 | 12.54 | N/A | N/A | 12.54 | 100 | 0 |
|  | 2 | 12.58 | 100 | 0.13 | 12.85 | 100 | 0.36 |
|  | 3 | 12.62 | 100 | 0.13 | 13.17 | 100 | 0.72 |
|  | 4 | 17.62 | 97.25 | 0.15 | 44.83 | 92.5 | 37.99 |
|  | 5 | 31.37 | 96.31 | 0.28 | 75.55 | 80 | 74.91 |
|  | 6 | 52.94 | 95.15 | 0.48 | 88.4 | 67.5 | 91.4 |
|  | 7 | 78.95 | 91.67 | 0.71* | 90.91 | 37.5 | 98.57 |
|  | 8 | 78.57 | 90.49 | 0.69 | 89.97 | 27.5 | 98.92 |
|  | 9 | 77.78 | 89.35 | 0.67 | 89.03 | 17.5 | 99.28 |
|  | 10 | 66.67 | 87.97 | 0.55 | 87.77 | 5 | 99.64 |
|  | 11 | 66.67 | 87.97 | 0.55 | 87.77 | 5 | 99.64 |
|  | 12 | N/A | 87.46 | N/A | 87.46 | 0 | 100 |
|  |  |  |  |  |  |  |  |
| NEWS | 1 | 13.13 | 95.45 | 0.09 | 18.81 | 97.5 | 7.53 |
|  | 2 | 13.48 | 94.59 | 0.08 | 22.88 | 95 | 12.54 |
|  | 3 | 15.97 | 97.53 | 0.14 | 36.68 | 95 | 28.32 |
|  | 4 | 18.13 | 96.03 | 0.14 | 48.9 | 87.5 | 43.37 |
|  | 5 | 22.15 | 95.88 | 0.18 | 61.44 | 82.5 | 58.42 |
|  | 6 | 33.72 | 95.28 | 0.29 | 78.68 | 72.5 | 79.57 |
|  | 7 | 42.31 | 93.26 | 0.36 | 84.95 | 55 | 89.25 |
|  | 8 | 66.67 | 92.47 | 0.59 | 90.28 | 45 | 96.77 |
|  | 9 | 64.29 | 89.84 | 0.54 | 88.71 | 22.5 | 98.21 |
|  | 10 | 75 | 89.07 | 0.64* | 88.71 | 15 | 99.28 |
|  | 11 | 71.43 | 88.78 | 0.6 | 88.4 | 12.5 | 99.28 |
|  | 12 | 66.67 | 88.5 | 0.55 | 88.09 | 10 | 99.28 |
|  | 13 | N/A | 87.46 | N/A | 87.46 | 0 | 100 |
|  |  |  |  |  |  |  |  |
| NEWS2 | 1 | 13.13 | 95.45 | 0.09 | 18.81 | 97.5 | 7.53 |
|  | 2 | 13.48 | 94.59 | 0.08 | 22.88 | 95 | 12.54 |
|  | 3 | 15.97 | 97.53 | 0.14 | 36.68 | 95 | 28.32 |
|  | 4 | 18.13 | 96.03 | 0.14 | 48.9 | 87.5 | 43.37 |
|  | 5 | 22.15 | 95.88 | 0.18 | 61.44 | 82.5 | 58.42 |
|  | 6 | 33.72 | 95.28 | 0.29 | 78.68 | 72.5 | 79.57 |
|  | 7 | 42.31 | 93.26 | 0.36 | 84.95 | 55 | 89.25 |
|  | 8 | 66.67 | 92.47 | 0.59 | 90.28 | 45 | 96.77 |
|  | 9 | 64.29 | 89.84 | 0.54 | 88.71 | 22.5 | 98.21 |
|  | 10 | 75 | 89.07 | 0.64* | 88.71 | 15 | 99.28 |
|  | 11 | 71.43 | 88.78 | 0.6 | 88.4 | 12.5 | 99.28 |
|  | 12 | 66.67 | 88.5 | 0.55 | 88.09 | 10 | 99.28 |
|  | 13 | N/A | 87.46 | N/A | 87.46 | 0 | 100 |
|  |  |  |  |  |  |  |  |
| HEWS | 1 | 13.93 | 97.44 | 0.11 | 24.14 | 97.5 | 13.62 |
|  | 2 | 18.78 | 97.54 | 0.16 | 48.9 | 92.5 | 42.65 |
|  | 3 | 27.27 | 96.46 | 0.24 | 70.22 | 82.5 | 68.46 |
|  | 4 | 33.77 | 94.21 | 0.28 | 79.62 | 65 | 81.72 |
|  | 5 | 48.65 | 92.2 | 0.41 | 87.15 | 45 | 93.19 |
|  | 6 | 68.18 | 91.58 | 0.6 | 89.97 | 37.5 | 97.49 |
|  | 7 | 76.92 | 90.2 | 0.67 | 89.66 | 25 | 98.92 |
|  | 8 | 80 | 89.64 | 0.7* | 89.34 | 20 | 99.28 |
|  | 9 | 71.43 | 88.78 | 0.6 | 88.4 | 12.5 | 99.28 |
|  | 10 | 66.67 | 87.97 | 0.55 | 87.77 | 5 | 99.64 |
|  | 11 | 50 | 87.7 | 0.38 | 87.46 | 2.5 | 99.64 |
|  | 12 | N/A | 87.46 | N/A | 87.46 | 0 | 100 |
|  |  |  |  |  |  |  |  |
| MEWS | 1 | 12.58 | 100 | 0.13 | 12.85 | 100 | 0.36 |
|  | 2 | 18.62 | 92.53 | 0.11 | 58.93 | 67.5 | 57.71 |
|  | 3 | 26.09 | 91.2 | 0.17 | 77.12 | 45 | 81.72 |
|  | 4 | 39.13 | 89.53 | 0.29 | 85.89 | 22.5 | 94.98 |
|  | 5 | 50 | 88.42 | 0.38* | 87.46 | 10 | 98.57 |
|  | 6 | 40 | 87.9 | 0.28 | 87.15 | 5 | 98.92 |
|  | 7 | 33.33 | 87.66 | 0.21 | 87.15 | 2.5 | 99.28 |
|  | 8 | 33.33 | 87.66 | 0.21 | 87.15 | 2.5 | 99.28 |
|  | 9 | N/A | 87.46 | N/A | 87.46 | 0 | 100 |
|  |  |  |  |  |  |  |  |

AUC: area under the curve of the Receiver Operating Characteristic; 95%C.I.: 95%Confidence Interval; Sen.: Sensitivity; Spe.: Specificity; PPV: positive predictive value; NPV: negative predictive value; LR+: likelihood ratio positive; LR-: likelihood ratio negative; NEWS: National Early Warning Score; NEWS2: National Early Warning Score2; HEWS: Hamilton Early Warning Score; MEWS: Modified Early Warning Score; SEWS: Standardized Early Warning Score; N/A: Not available because the denominator is zero

*largest Youden Index

**Supplementary 4**

**Results of the correlation between each EWS and SOFA^§^ (N=274)**

| **Models** | **Correlation coefficient of Spearman’s rank correlation test** | **p value** |
| --- | --- | --- |
| **SEWS** | 0.294 | 0.000 |
| **NEWS** | 0.330 | 0.000 |
| **NEWS2** | 0.330 | 0.000 |
| **HEWS** | 0.278 | 0.000 |
| **MEWS** | 0.221 | 0.001 |

EWS: Early Warning Score; SOFA: Sequential Organ Failure Assessment Scoring System ; NEWS: National Early Warning Score; NEWS2: National Early Warning Score2; HEWS: Hamilton Early Warning Score; MEWS: Modified Early Warning Score; SEWS: Standardized Early Warning Score;

**§**SOFA scores were calculated using worst value in 24 hours after patients' visit.

*p<0.05
